# Precise Short Sequence Insertion in Zebrafish Using a CRISPR/Cas9 Approach to Generate a Constitutively Soluble Lrp2 Protein

**DOI:** 10.3389/fcell.2019.00167

**Published:** 2019-08-13

**Authors:** Ross F. Collery, Brian A. Link

**Affiliations:** ^1^Department of Cell Biology, Neurobiology and Anatomy, Medical College of Wisconsin, Milwaukee, WI, United States; ^2^Department of Ophthalmology and Visual Sciences, Medical College of Wisconsin Eye Institute, Milwaukee, WI, United States

**Keywords:** zebrafish, CRISPR, gene editing, emmetropization, myopia

## Abstract

LRP2 is a large transmembrane receptor expressed on absorptive epithelia where it binds many extracellular ligands to control several signaling pathways. Mutations in *LRP2* are associated with buphthalmic eye enlargement, myopia and other non-ocular symptoms. Though studies have clearly shown that absence of LRP2 causes these phenotypes, and that overexpression of individual LRP2 domains can exacerbate eye enlargement caused by the absence of Lrp2, the relationship between soluble LRP2 fragments and full-length membrane-bound LRP2 is not completely understood. Here we use a CRISPR/Cas9 approach to insert a stop codon cassette into zebrafish *lrp2* to prematurely truncate the protein before its transmembrane domain while leaving the entire extracellular domain intact. The resulting mutant line will be a useful tool for examining Lrp2 function in the eye, and testing hypotheses regarding its extracellular processing.

## Introduction

Mutations in LRP2 are associated with Donnai-Barrow syndrome, whose symptoms include buphthalmic eye enlargement and myopia, as well as orbital hypertelorism, diaphragmatic hernia, agenesis of the corpus callosum, facial deformities, hearing loss, and intellectual disabilities (Donnai and Barrow, 1993; Kantarci et al., 2007, 2008; Pober et al., 2009). LRP2 is expressed in absorptive epithelia (Zheng et al., 1994; Lundgren et al., 1997), and binds many ligands associated with diverse signaling pathways, including Sonic Hedgehog (Shh), bone morphogenic protein (BMP), retinoid trafficking and others (Christensen et al., 1999; Christensen and Birn, 2002; Spoelgen et al., 2005; Christ et al., 2015). In the eye, LRP2 is expressed in the ciliary body and retinal pigment epithelium (RPE), where its absence causes dysregulation of eye size leading to myopia. Enlargement of the eye due to loss of LRP2 causes myopic retinopathy, with retinal ganglion cell damage and death due to stretch, and elevated intraocular pressure, reminiscent of glaucomatous phenotypes (Loyo-Berríos and Blustein, 2007; Veth et al., 2011; Cases et al., 2015). In the proximal tubules of the kidney, LRP2 is associated with binding and recovery of proteins prior to excretion, and its absence leads to proteinuria (Kantarci et al., 2007). In the kidney, LRP2 has been shown to be processed by regulated intramembrane proteolysis (RIP), where the C-terminal domain is cleaved at a γ-secretase site before entering the nucleus to regulate gene expression (Zou et al., 2004; Li et al., 2008). Multiple *Lrp2* mutant mouse lines have been generated that show enlarged eyes, but have inconsistencies in other phenotypes – one line shows high lethality while the other does not (Zarbalis et al., 2004; Spoelgen et al., 2005). We have recently demonstrated that extracellular cleavage of Lrp2 to release a ligand-binding domain may function as a switch, converting the membrane-bound endocytic receptor to a soluble decoy that alters signaling by bound ligands (Collery and Link, 2018). In the eye, soluble N-terminal domains of Lrp2 expressed from the RPE lead to eye enlargement and myopia similar to that seen in *lrp2* mutant zebrafish. By inhibiting proper signaling through membrane-bound Lrp2, both *lrp2*^–/–^ animals (no signaling facilitated) and animals overexpressing soluble Lrp2 (soluble domain binds ligands and prevents interaction with membrane-bound receptors) exhibit a large-eyed phenotype. Uncovering how extracellular cleavage of LRP2 is regulated will be vital to understanding the nature of its effects on eye size and emmetropization.

CRISPR/Cas9 technology has become a vital tool for precise gene editing despite its short history (Doudna and Charpentier, 2014). Tools targeting genes of interest can be synthesized quickly and easily by researchers, or purchased commercially. In the zebrafish community, the CRISPR/Cas9 system has been rapidly adopted to great effect. Along with zinc-finger nucleases (ZFNs) and transcription activator-like effector nucleases (TALENs), CRISPRs have provided zebrafish researchers with the opportunity to accurately target genes for deletion, tagging or editing (Hwang et al., 2013; Jao et al., 2013). Though zebrafish have long been promoted for their ease of transgenesis and transparent *ex vivo* development facilitating real-time imaging of fluorescent proteins (Kawakami, 2004; Mosimann et al., 2013; Kawakami et al., 2016), precise gene editing in zebrafish has lagged behind that of other model organisms – though many excellent forward genetic screens using randomly acting mutagenic agents have been undertaken, including screens focusing on eye development and function (Haffter et al., 1996; Fadool et al., 1997; Amsterdam et al., 1999; Wienholds et al., 2003; Gross et al., 2005; Muto et al., 2005; Lee et al., 2012). Application of CRISPR/Cas9 tools allow programing of the guide RNA to genomic regions by use of a (∼20 nucleotide sequence followed by an invariant *trans-*activating CRISPR RNA that recruits the Cas9 protein for DNA cutting. Following cutting, DNA is rapidly repaired either by non-homologous end-joining, leading to frequent insertions of deletions (indels) that can disrupt inframe translation of targeted protein-coding genes, or by homology-directed repair, where an exogenous DNA template provide the homology necessary for precise repair of a double-strand break. By combining CRISPR/Cas9 with a DNA template containing homology arms flanking an exogenous sequence, precise genomic editing can be used to insert an epitope tag, selectively edit individual codons, or mutate transcription-factor binding sites (Gagnon et al., 2014). In zebrafish, design of single-stranded oligodeoxynucleotides (ssODNs) containing stop codons in multiple reading frames with 20 nt homology flanks have been described, which are combined with CRISPRs to inactivate protein translation while relieving the need for inframe integration of the stop codon (Gagnon et al., 2014). Using a similar approach, we designed a CRISPR immediately upstream of the zebrafish Lrp2 transmembrane domain, and an ssODN containing stop codons in all three reading frames (3xSTOP) with 20 nt homology arms. With this approach, we set out to generate a mutant zebrafish line that would express all extracellular domains of Lrp2 while being exclusively soluble owing to its lack of transmembrane or intracellular domains. Here we report that CRISPR/Cas9 editing of the zebrafish *lrp2* coding region led to precise in-frame insertion of a short DNA fragment as intended, resulting in a predicted c.S4424N^*^ Lrp2 protein. *lrp2*^S4424N*/S4424N*^ zebrafish homozygotes displayed enlarged, myopic eyes similar to *lrp2*C23X/C23X mutants described earlier. This new mutant line will facilitate research into Lrp2 processing, as well as testing hypotheses involving factors linked to Lrp2 interaction and refractive error, such as Bmp4 or Shh.

## Materials and Methods

### Zebrafish Husbandry

Zebrafish (*Danio rerio*) were maintained using standard methods (Westerfield, 2007). All animal husbandry and experiments were approved and conducted in accordance with the guidelines set forth by the Institutional Animal Care and Use Committee of the Medical College of Wisconsin.

### CRISPR/Cas9 Generation and Application

**FIGURE 1:**
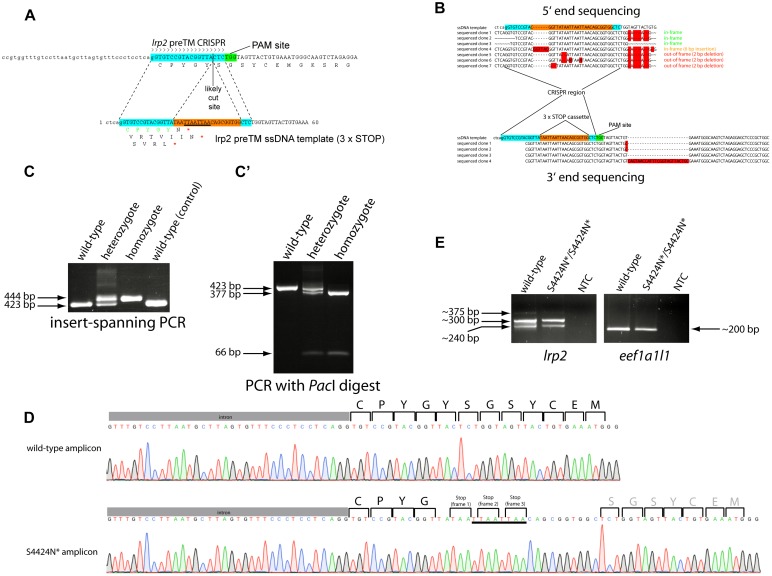
**(A)** Schematic showing the zebrafish *lrp2* genomic region upstream of the transmembrane domain with CRISPR target site. The 19 nt target site is highlighted in turquoise while the adjacent protospacer adjacent motif (PAM) site is shown in green. The most likely Cas9 cut site is shown 3 nt 5′ from the PAM site. The ssODN template used to introduce the 3xSTOP cassette is shown with the CRISPR target site split by the cassette (orange). The *Pac*I restriction site contained in the cassette is underlined. **(B)** 5′ and 3′ end sequencing of germline integrations of the 3xSTOP cassette show several inframe insertions, as well as others that are out of frame. **(C)** After establishing the *lrp2* S4424N^*^ line, PCR and/or restriction enzyme digest can be used to easily track the insertion and assess the zygosity. A high percentage agarose gel can resolve the 20 bp insertion from wild-type, while restriction fragment length polymorphism can also confirm the genotype **(C’)**. **(D)** Sanger sequencing traces of wild-type and *lrp2* S4424N^*^ homozygous DNA show the presence of stop codons in all three reading frames in the mutant. **(E)** RT-PCR showed *lrp2* mRNA species present in wild-type and S4424N^*^ homozygotes at the expected size (298 bp). Other bands likely indicate splice variants.

Guide RNAs targeting the *lrp2* pre-transmembrane domain were designed using the ZiFiT Targeter software package (Sander et al., 2007, 2010). The genomic region immediately upstream of the coding sequence for the Lrp2 transmembrane domain was queried for suitable targeting sites, before selecting a 19 bp site in exon 74, GGTGTCCGTACGGTTAC TCTGG, where Cas9 cutting is predicted to occur between the two underlined nucleotides, and the PAM site is highlighted in bold font (see Figure 1). During CRISPR design, potential off-target regions were noted, and the relevant regions located in coding sequences were amplified by PCR for sequencing. Oligonucleotides (see Table 1) were used to clone the target sequence into pDR274 [a gift from Keith Joung (Addgene plasmid # 42250)] to be used as a template for *in vitro* transcription of the guide RNA with customized *lrp2*-targeting sequence included (Hwang et al., 2013). The MEGAshortscript T7 transcription kit (Ambion, Foster City, CA, United States) used to synthesize guide RNA, which was purified using a mirVana miRNA Isolation Kit (Ambion). Guide RNA was combined with *in vitro*-transcribed zebrafish codon-optimized *Cas9* mRNA with nuclear-localization signals (Jao et al., 2013), and a ssODN containing a 3xSTOP cassette flanked by 20 nucleotides complementary to the *lrp2* genomic sequence (Gagnon et al., 2014). The CRISPR/Cas9/ssODN mix was injected into 1 to 4-cell ZDR wild-type zebrafish eggs and allowed to develop normally. Injected fish were bred to assess their offspring for germline transmission of the 3xSTOP cassette, as well as for perfect in-frame integration.

**TABLE 1:** Oligonucleotides used in this work.

**Name**	**Sequence (5′ – 3′)**
lrp2preTMCRISP1-F1	TAGGTGTCCGTACGGTTACTC
lrp2preTMCRISP1-R1	AAACGAGTAACCGTACGGACA
lrp2 3xSTOP ssODN	ctcagGTGTCCGTACGGTTATAATTAATTAACA
	GCGGTGGCTCTGGTAGTTACTGTGAAA
lrp2-mRNA-F1	GGCAGTTTACTTGCATGAATGGCCGC
lrp2-mRNA-R1	TTTGGGTCGCAGGGTCTGAAGATGC
eef1a1l1ex1-2-F	TCTCTCAATCTTGAAACTTATCAATCA
eef1a1l1ex3-R	AACACCCAGGCGTACTTGAA

### DNA Extraction and Analysis

Genomic DNA was extracted from pooled larvae or adult finclip samples using the Qiagen Gentra Puregene Tissue Kit (Qiagen, Germantown, MD, United States). PCR was carried out using the AccuPrime^TM^ *Taq* DNA Polymerase System (Thermo Fisher Scientific, Waltham, MA, United States), with oligonucleotides synthesized by Integrated DNA Technologies (IDT, Coralville, IA, United States). *Pac*I restriction enzyme was supplied by New England Biolabs (NEB, Ipswich, MA, United States). All oligonucleotide sequences are provided in Table 1.

### RNA Extraction, cDNA Synthesis and Reverse Transcription-PCR

RNA was extracted using TRIzol (Thermo Fisher Scientific, Waltham, MA, United States). 100 ng of RNA was DNaseI-treated and cDNA synthesized using the SuperScript III kit (Thermo Fisher Scientific, Waltham, MA, United States). RT-PCR was carried out using intron-spanning *lrp2* primers and eukaryotic translation elongation factor 1 alpha 1-like 1 (*eef1a1l1*) primers as controls (Skarie and Link, 2008).

### Mini-Gene Cloning and Overexpression in HEK Cells

Zebrafish DNA was cloned into the Tol2 Gateway system for overexpression studies in cell culture (Kwan et al., 2007). HEK293T cells were transfected with plasmid DNA and cultured for 36 h. Proteins were separated on Bio-Rad Any kD gradient SDS-PAGE gels before transferring to Immobilon-F PVDF membranes. Western blots were probed using anti-eGFP antibody JL-8 (Clontech, Mountain View, CA, United States) and developed using the LI-COR Odyssey buffer and imaging system (LI-COR, Lincoln, NE, United States).

### Spectral Domain-Optical Coherence Tomography

Zebrafish eyes were imaged using a Bioptigen Envisu R2200 SD-OCT imaging system with a 12 mm telecentric lens (Bioptigen, Morrisville, NC, United States) as previously described (Collery et al., 2014). Relative refractive errors (RREs) were calculated using the formula 1- (retinal radius/F), where F, an idealized focal length = lens radius × 2.324, a previously determined constant. To assess the effect on eye metrics and refractive error following genomic editing, each experiment was conducted three separate times, with a minimum of eight eyes per genotype per experiment without left-right eye bias.

### Statistical Analyses

Eye measurements were processed using Microsoft Excel (Microsoft, Redmond, WA, United States) and graphed using GraphPad Prism (GraphPad, La Jolla, CA, United States). Standard deviation (SD) and ordinary analysis of variance (ANOVA) with Tukey’s multiple comparisons post-test analysis were calculated using GraphPad Prism.

## Results

### CRISPR/Cas9 Methods Combined With ssODN HDR Templating Allow Perfect Short Sequence Integration

A CRISPR guide RNA was designed that targeted the genomic region immediately upstream of the zebrafish *lrp2* transmembrane domain using the ZiFit program (Sander et al., 2007, 2010). Using the approach of Gagnon et al. (2014), an ssODN was designed containing stop codons in all three reading frames (3xSTOP) flanked by 20 nt homology arms corresponding to regions immediately surrounding the likely CRISPR cut site (Figure 1A). The 3xSTOP cassette contains a *Pac*I restriction enzyme cut site to assist identification and tracking of zebrafish carrying this insert. The CRISPR guide, ssODN and Cas9 mRNA were injected into 1-2-cell stage wild-type embryos. Coding sequences for genes containing more than 12 nt homology with the *lrp2*-targeting CRISPR were sequenced to verify that no off-target genomic editing took place (Supplementary Figure S1). Specifically, coding sequences from *adamts12, C1GALT1-specific chaperone 1*, and *wdr32* were verified as being unchanged from reference sequences, since they contained some similarity to the CRISPR target site. The 3′ untranslated region from *zinc finger protein 271-like* was also examined, and found to contain a single C > T nucleotide change that would not affect the amino acid sequence. After reaching maturity, injected fish were outcrossed to wild-type fish, and pools of their offspring were screened by PCR for successful integration of the 3xSTOP cassette using one primer internal to the cassette and one primer complementary to the local genomic region. Following successful identification of positive founder parents, the remaining embryos from these or repeat crosses were raised to adulthood. Putative F1 carriers of the 3xSTOP insertion were genotyped by finclip and subsequent PCR as before. PCR amplicons from positive individuals were TOPO-cloned for both 5′ and 3′ ends. Sequencing confirmed a high number of individual fish carrying insertions that faithfully incorporated the 3xSTOP cassette into the zebrafish genome while maintaining the sequence templated by the ssODN. We note, however, that several individuals were found containing 3xSTOP insertions that were incorporated out of the correct reading frame; either by incorporating extraneous 5′ sequence, extraneous 3′ sequence, or by corrupting the cassette itself (Figure 1B).

Adult F2 zebrafish identified as heterozygous for the 3xSTOP cassette with perfect base-pair sequence were incrossed. Wild-type, heterozygote and homozygous zebrafish could all be easily distinguished by agarose gel electrophoresis both by PCR alone, and also by using *Pac*I digestion to confirm the presence of the cassette (Figures 1C,C’). Due to the premature stop(s) caused by the integrated cassette, the protein sequence of Lrp2 is shortened from 4673 amino acids to 4424, with the amino acid before the first stop codon changed from S to N. This allele is therefore named *lrp2*^S4424N*^. Symbols “X” and “^*^” are both used to show a stop codon in a protein sequence. Genomic sequencing shows homozygous mutant DNA carrying the intended edit (Figure 1D).

**FIGURE 2:**
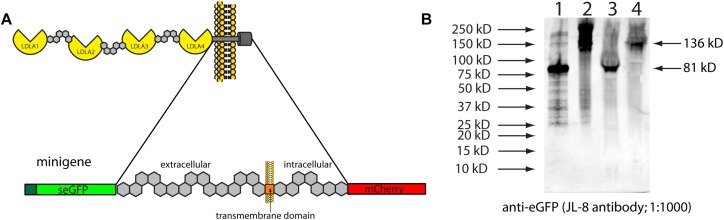
**(A)** Schematic showing the zebrafish Lrp2 minigene containing the partial extracellular region, and the complete transmembrane and intracellular regions. The minigene is flanked by seGFP and mCherry, which adds size discrimination masses, and facilitates protein detection. **(B)** HEK293T cells transfected with plasmids expressing minigene variants under the control of the CMV promoter. Protein extracts were Western blotted and probed using anti-eGFP (JL-8; Clontech). Key: (1) seGFP-Lrp2 ECD-STOP-ICD-mCherry; (2) seGFP-Lrp2 ECD-readthrough cassette-ICD-mCherry. Similar results were seen when plasmids were injected into wild-type zebrafish embryos.

To verify *in vivo* that inserting stop codons in this location would lead to the predicted truncation, we cloned part of zebrafish *lrp2* into overexpression plasmids. We used this “minigene” approach due to the extremely large size of full-length *lrp2*, which makes cloning difficult. DNA corresponding to 691 amino acids of zebrafish *lrp2* was amplified for cloning; this corresponded to the region surrounding the transmembrane domain, where the zebrafish genomic 3xSTOP cassette was inserted (Figure 2). The cloned region, approximately 15% of full-length Lrp2, was flanked with secreted eGFP at the N-terminus, and mCherry at the C-terminus, and placed under the control of a CMV promoter. The resulting plasmid was named *pTol2-CMV:SP6-seGFP-lrp2 ECD-ICD-mCherry*. At the region immediately upstream of the transmembrane domain, the plasmid was edited to contain one of the following short sequences: 1. a stop codon to mimic zebrafish genomic *lrp2* following insertion of the stop cassette; 2. a DNA sequence of similar length to that inserted into the zebrafish genome, but coding for an inert amino acid sequence that allows readthrough (LGAIQAQQRVRNRFA).

Western blotting results showed that insertion of a stop codon in the equivalent location in cloned mini-*lrp2* led to premature termination, and no inclusion of the transmembrane domain or other downstream domains (∼81 kD). In contrast, the plasmid containing cloned mini-*lrp2* with no stop codon led to translation of the full construct, and included transmembrane domain, intracellular domain, and mCherry (∼135 kD). Similar results were seen following injection of the same plasmids into wild-type zebrafish.

### *lrp2*^S4424N*/S4424N*^ Homozygous Fish Show Persistent *lrp2* mRNA Without Nonsense-Mediated Decay

In order to assess whether *lrp2* mRNA was present at normal levels following 3xSTOP cassette integration, we extracted total RNA from wild-type and *lrp2*^S4424N*/S4424N*^ eyes. RT-PCR using intron-spanning primers unique to *lrp2* showed similar levels of *lrp2* transcript, as well as similar levels of *eef1a1l1* as a control (Figure 1E). Two major bands were apparent, likely representing the inclusion of an exon in the larger band (Supplementary Figure S2).

### *lrp2*^S4424N*/S4424N*^ Homozygous Fish Have Enlarged Eyes With Occasional Unilateral Asymmetry

Gross morphological analyses of wild-type, heterozygous and homozygous *lrp2*^S4424N*/S4424N*^ head and eyes was carried out as previously performed (Veth et al., 2011). To compare existing *lrp2* null mutants with novel lines expressing full-length extracellular Lrp2, we imaged wild-type fish, *lrp2*^mw1^ (C23X) and *lrp2*^S4424N*/S4424N*^.

**FIGURE 3:**
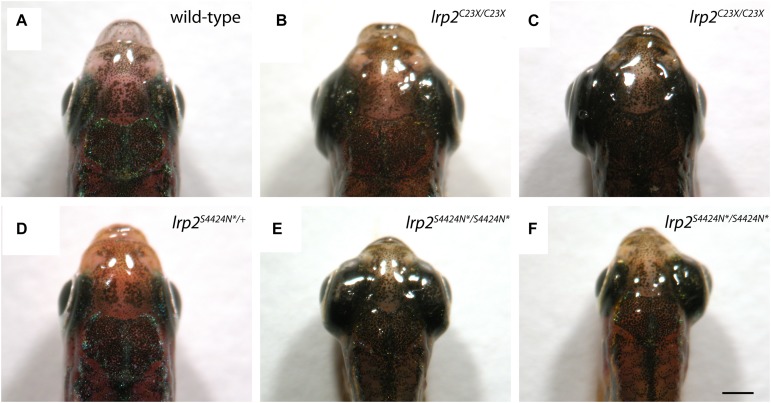
**(A)** Gross dorsal morphology of wild-type zebrafish shows streamlined eyes lying close to the contours of the head. **(B)** Homozygous *lrp2* C23X mutant zebrafish documented by Veth et al. (2011), and others, show buphthalmic eye enlargement. **(C)** Occasional unilateral eye enlargement is seen in the C23X allele. **(D)** Heterozygous zebrafish with one *lrp2* S4424N^*^ allele and one wild-type allele are similar to wild-type siblings and show no eye enlargement. **(E)** Homozygous zebrafish carrying two S4424N^*^ alleles leading to premature protein truncation upstream of the Lrp2 transmembrane domain show buphthalmic eye enlargement similar to C23X homozygotes. **(F)** Occasional unilateral eye enlargement was seen in S4424N^*^/S4424N^*^ homozygotes, similar to C23X homozygotes. Scale bar = 1 mm.

Wild-type adult zebrafish have eyes that sit close to the head evolved for streamlined movement through water (Figure 3A). Previously published work shows that zebrafish mutants containing a premature stop codon at residue 23 exhibit buphthalmic eyes, which are most often similarly enlarged (Figure 3B), but can sometimes be unilateral (Figure 3C). Heterozygous zebrafish with a single S4424N^*^ allele are indistinguishable from wild-type fish (Figure 3D). However, homozygous fish with S4424N^*^ at both alleles exhibited high buphthalmia, with frequent bilateral enlargement (Figure 3E). Similar to *lrp2*^mw1^ (C23X), unilateral eye enlargement was sometimes observed (Figure 3F). We note that homozygous S4424N^*^ mutants obtained from the first generation of heterozygotes used for inbreeding always had eyes larger than wild-types or heterozygotes, contrasting with *lrp2*^mw1^ (C23X) which showed reduced phenotypic penetrance for several generations after identification (Veth et al., 2011).

### *lrp2*^S4424N*/S4424N*^ Homozygous Fish Are Myopic

We have previously established a method of acquiring accurate *in vivo* measurements of zebrafish eye metrics using SD-OCT (Collery et al., 2014). We can use these eye metrics to calculate the RRE of each eye as a measure of deviation from a hypothetical perfectly focused eye. In addition, we can also normalize for eye size using the length of the fish body as a denominator to express the eye size as a function of overall size. Cohorts of sibling fish *lrp2*^S4424N*/+^ heterozygote incrosses were genotyped and their eye metrics measured at 2 months of age.

**FIGURE 4:**
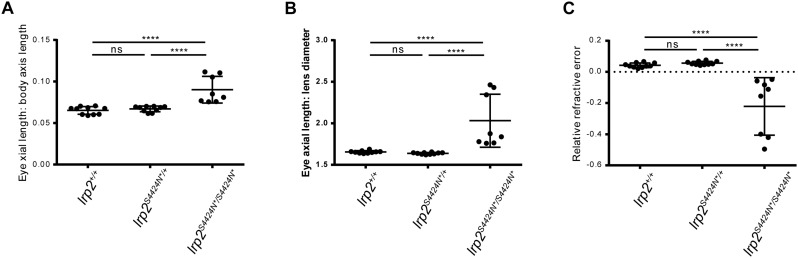
**(A)** Eye axial length: body axis ratios show that wild-type and heterozygous *lrp2* S4424N^*^ have similar means and distributions, while *lrp2* S4424N^*^ homozygotes show a higher mean with a wider distribution, indication that eyes in these fish are larger when normalized using body axis as an independent metric. **(B)** Similarly, when using the lens as a normalizing metric internal to the eye, *lrp2* S4424N^*^ homozygote axial lengths are greater than heterozygotes or wild-types. **(C)** Relative refractive errors (a measure of the deviation of an eye from perfect focus) of wild-type and heterozygous *lrp2* S4424N^*^ eyes are both slightly hyperopic, while *lrp2* S4424N^*^ homozygote eyes are very myopic. Genotypes were analyzed for significance using an ordinary one-way ANOVA with Tukey’s multiple comparisons post-test. ^∗∗∗^*p* < 0.0001; ns, not significant.

Using body length to normalize between individuals, wild-type zebrafish showed an average eye axial length: body axis ratio of 0.065 (±0.0046 SD) (Figure 4A). Similarly, heterozygous S4424N^*^/+ fish showed an average ratio of 0.067 (±0.0046 SD). No significant difference was seen between these two groups by one-way ANOVA. However, when homozygous S4424N^*^/S4424N^*^ zebrafish were measured, their eye axial length: body length ratio was 0.09 (±0.016 SD), which was very significantly different from the other two groups (*p* < 0.0001 by one-way ANOVA).

Using the lens as a metric internal to the eye that we assume to be unaffected by factors affecting axial length growth, wild-type, heterozygous and homozygous fish were compared. Wild-type zebrafish showed an average eye axial length: lens diameter ratio of 1.655 (±0.015 SD) (Figure 4B). Similarly, heterozygous S4424N^*^/+ fish showed an average ratio of 1.638 (±0.011 SD). No significant difference was seen between these two groups by one-way ANOVA. However, when homozygous S4424N^*^/S4424N^*^ zebrafish were measured, their eye axial length: lens diameter ratio was 2.032 (±0.320 SD), which was very significantly different from the other two groups (*p* < 0.0001 by one-way ANOVA).

Using eye axial length, lens diameter and retinal radius to calculate the RRE of the three groups, wild-type and heterozygous fish were very slightly hyperopic, having mean RREs of 0.038 (±0.014 SD) and 0.056 (±0.012 SD), respectively, where a measure of 0 indicates a perfectly focused eye, positive values indicate hyperopia, and negative values indicate myopia (Figure 4C). No significant difference was seen between these two groups by one-way AVOVA. However, homozygous S4424N^*^/S4424N^*^ fish had a mean RRE of −0.222 (±0.184), indicating that these fish are myopic. The homozygous group differed significantly from the two control groups (*p* < 0.0001 by one-way ANOVA). Taken together, these data indicate that the presence of exclusively soluble Lrp2 leads to eye enlargement and myopia.

### *lrp2*^S4424N*/S4424N*^ Homozygous Fish Have Anterior Segment Changes Similar to *lrp2*^C23X/C23X^ Fish

**FIGURE 5:**
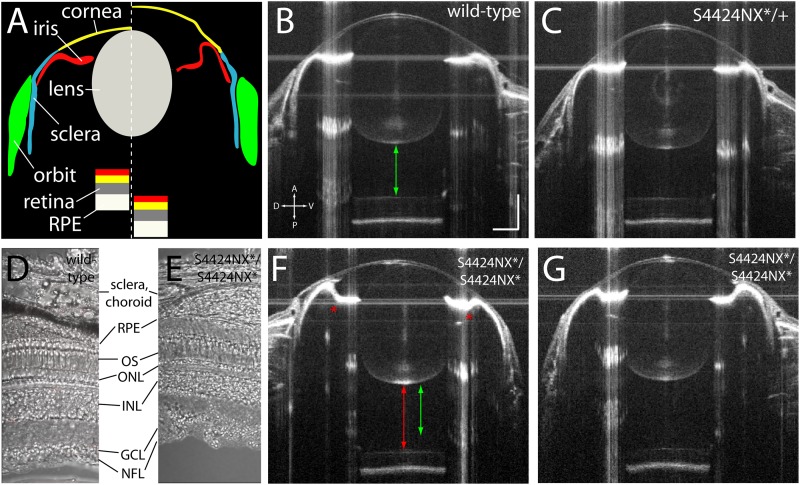
**(A)** Schematic showing wild-type (left of dashed line) and *lrp2* mutant eye morphology (right of dashed line) as visualized by OCT. In *lrp2* mutant eyes the retina is further away from the lens, and there is a larger gap between lens and cornea. Note the concavity of the iris in the mutant eye. **(B)** 2 mpf wild-type eye. Green arrow shows the distance from the back of the lens to the front of the retina, a proxy for vitreous chamber depth. Scale bar = 300 μm with the same scale used throughout the figure. **(C)** *lrp2* S4424N^*^/+ heterozygous eye shows similar metrics to the wild-type eye. **(D,E)** Histological sections show normal lamination of wild-type and S4424NX^*^ homozygous eyes (RPE, retinal pigment epithelium; OS, outer segments; ONL, outer nuclear layer; INL, inner nuclear layer; GCL, ganglion cell layer; NFL, nerve fiber layer). **(F,G)** *lrp2* S4424N^*^/S4424N^*^ homozygous eyes (left and right, respectively) show enlargement with glaucomatous phenotypes. The iris bulges inward (asterisk) and the vitreous chamber depth (red arrow) is greater than in the sibling wild-type from panel **(B)** (green arrow).

Acquisition of biometric data for measurement by SD-OCT also allows visualization of the anatomy of the eye. SD-OCT imaging allows the anterior (cornea, iris, lens, aqueous) and posterior (retina, RPE, vitreous) parts of the eye to be viewed *in vivo* (Figure 5A). Wild-type and heterozygous S4424N^*^/+ show of wild-type, heterozygous and homozygous S4424N^*^/S4424N^*^ show normal cornea and lens morphologies with near-planar irises (Figures 5B,C). Laminated retinas with clearly visible retinal ganglion cell layers and highly reflective RPEs could be clearly discerned. Histological analysis of dissected retinas from wild-type and S4424NX^*^/S4424NX^*^ eyes show normal lamination (Figures 5D,E), and no overt differences in the retinas, choroids or scleras were observed in mutant eyes. While homozygous S4424N^*^/S4424N^*^ eyes also had normal corneal and lenticular anatomies, the irises were frequently concave rather than planar, and accompanied by deepening of the anterior chamber (Figures 5F,G). Extreme concavity of the iris was occasionally seen in the most enlarged eyes. This likely corresponds to the ciliary epithelial hypertrophy previously observed in *lrp2* C23X/C23X zebrafish eyes (Veth et al., 2011).

## Conclusion

Extracellular cleavage of LRP-1 protein has been well documented (Herz et al., 1988, 1990), and we have proposed that LRP2 is also cleaved to yield a soluble extracellular form, allowing LRP2 to act as a switch between membrane-bound endocytosis and soluble decoy (Collery and Link, 2018). Since the factor(s) that carry out cleavage of LRP2 are currently unknown, we elected to use CRISPR/Cas9 methods to generate a constitutively soluble version of Lrp2 in zebrafish by inserting a short cassette containing stop codons into the coding sequence just before the transmembrane domain.

We combined an *lrp2*-targeting CRISPR with an ssODN containing the 3xSTOP cassette flanked by short homology arms (Gagnon et al., 2014). The cassette was designed to contain stop codons in all three reading frames so that even imperfect integration would force protein translation to terminate. We demonstrate that this approach is suitable for making small insertions into native gene loci in order to test hypotheses (premature truncation or insertion of proteolytic cleavage site), epitope tagging of endogenous proteins, or directing a protein toward a novel location using a signal peptide or transmembrane domain. Combining validated CRISPR guides with commercially available ssODNs allows rapid and cost-effective HDR priming, without the need for expensive and tedious generation of long, double-stranded templates.

After injection of the CRISPR/Cas9/ssODN mixture, several founder fish were identified by PCR that transmitted the 3xSTOP cassette to their offspring. Subsequent sequencing of the insertion site showed that while perfect inframe integration was present in a number of families, out-of-frame insertions were also present in others. Though the cassette should be effective in each frame, we chose to discard the out-of-frame insertion families and to focus only on the insertion that integrated without errors. In cases where the insertion contained an epitope tag, or other sequence relying on maintenance of the proper reading frame, this approach would be vital. We also feel that the potential for out-of-frame integrations emphasizes the importance of vigilant sequencing of genomic DNA, rather than only relying on the presence of a PCR amplicon.

After identifying several individual adult zebrafish heterozygous for the 3xSTOP cassette, incrossing of those fish yielded families of fish containing homozygotes, heterozygotes and wild-types. Comparing groups of fish from individual families controls for the presence of SNPs or other unknown sequence variants, and segregated fish are unlikely to have substantially diverse backgrounds, differing only by their 3xSTOP status. We noted that insertion of the 3xSTOP cassette did not lead to nonsense-mediated decay of mRNA, indicating that the truncated Lrp2 protein is likely present. Using a minigene approach, we verified that insertion of a stop codon in this region leads to premature protein translation as predicted. Use of this minigene approach was necessary since, despite our best efforts, we have been unable to use antibody-based methods (or indeed, any other method) to detect wild-type or truncated Lrp2. We note that the minigene contains over 15% of the total Lrp2 coding region, with more than 400 amino acids N-terminal to the premature stop site, and almost 300 amino acids C-terminal; though this assay is a proxy for observing premature truncation of full-length Lrp2 expressed from its native locus, we propose that it adequately represents molecular events that take place.

We note that in contrast to initial studies of zebrafish *lrp2* mutants, which exhibited low penetrance for multiple generations, our S4424N^*^/S4424N^*^ homozygotes showed consistent eye enlargement phenotypes immediately (Veth et al., 2011). We consider that this may be due to a number of factors, including a greater effect on emmetropic signaling caused by predicted constitutively soluble Lrp2 acting in a dominant negative manner; a greater degree of background homogeneity in the wild-type line used in this study reducing the likelihood of unknown modifier alleles; or better resolution of measurement associated with using OCT rather than simple observation to acquire eye metrics.

Wild-type and heterozygous S4424N^*^/+ adult fish showed normal eye sizes and slight hyperopia at 2 mpf, similar to earlier reports on fish of this age (Collery et al., 2014). However, S4424N^*^/S4424N^*^ homozygotes showed frequent buphthalmic eye enlargement and high myopia, comparable to the *lrp2/bugeye* phenotype. Our conclusion is that in a series of experiments well-controlled for genetic background as well as variability between individuals, the conversion of Lrp2 from a full-length membrane-bound form subject to potential regulated extracellular cleavage to a constitutively soluble decoy form leads to extreme eye enlargement and myopia.

Due to the similarities between *lrp2* S4424N^*^/S4424N^*^ and C23X/C23X homozygous zebrafish eyes, which share phenotypic changes including eye enlargement, myopia, and ciliary epithelial hypoplasia, it is likely that the mechanism that causes these phenotypes is the same. As detailed in our earlier work (Collery and Link, 2018), we propose that absence of Lrp2 protein or excessive levels of soluble Lrp2 domains have a similar effect on the eye, causing a reduction in proper emmetropic signaling. In both cases, membrane-bound Lrp2 is not present to facilitate normal regulation of eye size, and buphthalmia is the result. Though we have not repeated all phenotyping assays detailed by Veth et al. (2011), such as intraocular pressure measurement, and immunomorphological profiling, we believe it is likely that both mutant lines would show similar findings.

Future work will include a complementarity assay, where we examine transheterozygous zebrafish carrying one C23X allele and one S4424N^*^ allele. In this way, we can assess whether the absence of Lrp2, or the presence of full-length soluble Lrp2 leads to eye enlargement and myopia via the same mechanism. We can also use the S4424N^*^ line to study soluble Lrp2 interactions with Bmp and Shh pathway components. We will combine our transgenic zebrafish line *Tg(rpe65a:Gal4 UAS:LDLA1-eGFP)*, which increases the severity of *lrp2* C23X myopia, with the *lrp2* S4424N^*^ line to see if the effect is the same. In addition, we will use the CRISP-Cas9/ssODN approach detailed here to insert epitope tags into Lrp2 to track the location of its domains, and to study protein–protein interactions.

## Ethics Statement

All animal husbandry and experiments were approved and conducted in accordance with the guidelines set forth by the Institutional Animal Care and Use Committee of the Medical College of Wisconsin.

## Author Contributions

RC conceived and executed the experiments, and wrote the manuscript. BL proofread the manuscript and made helpful suggestions regarding both experiments and manuscript preparation.

## Conflict of Interest Statement

The authors declare that the research was conducted in the absence of any commercial or financial relationships that could be construed as a potential conflict of interest.

## Supplementary Material

FIGURE S1 Genomic sequencing of coding sequences candidates for off-target CRISPR effects based on sequence homology show no deviation from the reference genome. **(A)** Target sequence of zebrafish *lrp2*-targeting CRISPR. **(B–D)** Coding sequences for *adamts12, C1GALT1-specific chaperone 1*, and *wdr32* are all unchanged with respect to published sequences. **(E)** 3′ untranslated region for *zinc finger protein 271-like* has a single C > T change; this is unlikely to affect the translated protein or its function.

FIGURE S2 **(A)** Ensembl graphic showing alignment of *lrp2* RT-PCR amplicon (hatched red blocks) aligned with zebrafish genomic build GRCz11. Dashed green box indicated exon 74 which is targeted for CRISP/Cas9 editing in this work. Black box indicates exon 73, which may not be spliced into all transcripts, and is not annotated in EST libraries. The higher band see in RT-PCR corresponds to the inclusion of exon 73, while the lower band does not contain this exon. **(B)** Sequence trace image shows the boundaries of exon 72–73 and exon 73–74 splicing (exon 73 marked in blue). **(C)** A sequence trace lacking exon 73 represents alternate splicing.
