# Supplementary figures and images for: Precise Short Sequence Insertion in Zebrafish Using a CRISPR/Cas9 Approach to Generate a Constitutively Soluble Lrp2 Protein
Source: Front Cell Dev Biol. 2019 Aug 13;7:167. doi: 10.3389/fcell.2019.00167 (PMC6700241; doi:10.3389/fcell.2019.00167)

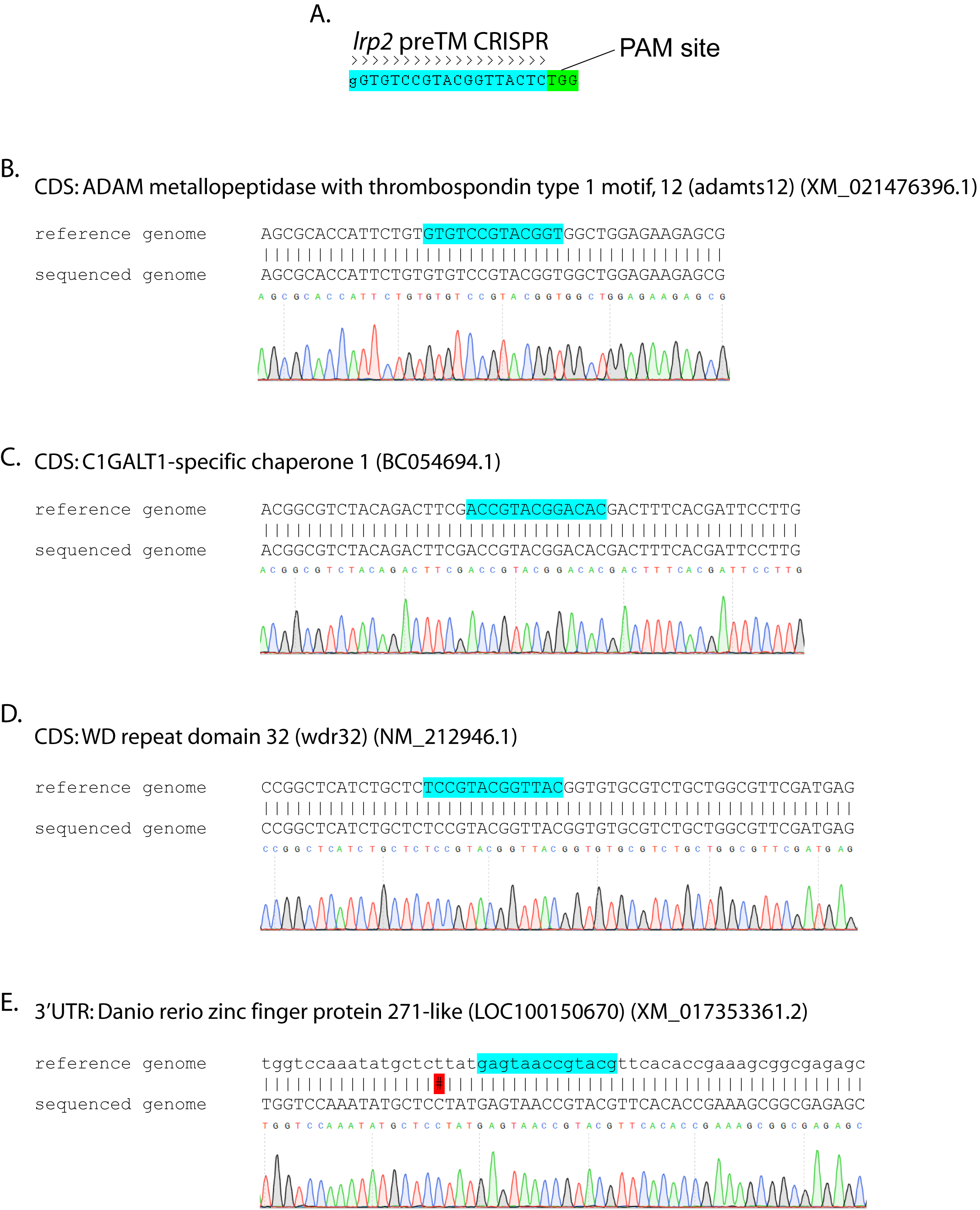

Supplement: FIGURE S1 — Genomic sequencing of coding sequences candidates for off-target CRISPR effects based on sequence homology show no deviation from the reference genome. (A) Target sequence of zebrafish lrp2-targeting CRISPR. (B–D) Coding sequences for adamts12, C1GALT1-specific chaperone 1, and wdr32 are all unchanged with respect to published sequences. (E) 3′ untranslated region for zinc finger protein 271-like has a single C > T change; this is unlikely to affect the translated protein or its function. [file Image_1.TIF]

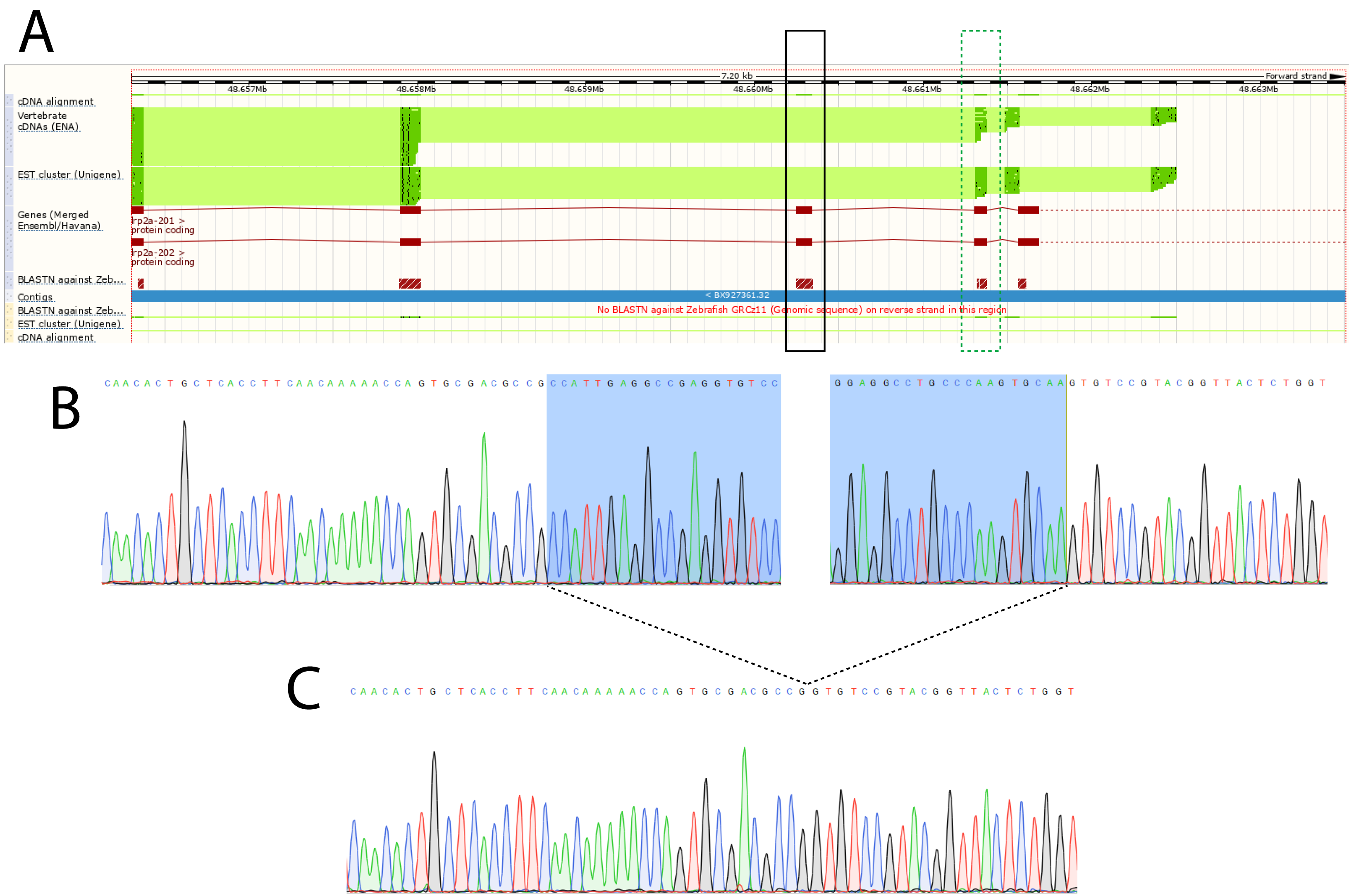

Supplement: FIGURE S2 — (A) Ensembl graphic showing alignment of lrp2 RT-PCR amplicon (hatched red blocks) aligned with zebrafish genomic build GRCz11. Dashed green box indicated exon 74 which is targeted for CRISP/Cas9 editing in this work. Black box indicates exon 73, which may not be spliced into all transcripts, and is not annotated in EST libraries. The higher band see in RT-PCR corresponds to the inclusion of exon 73, while the lower band does not contain this exon. (B) Sequence trace image shows the boundaries of exon 72–73 and exon 73–74 splicing (exon 73 marked in blue). (C) A sequence trace lacking exon 73 represents alternate splicing. [file Image_2.TIF]
